# Antiproteinuric effect of add-on paricalcitol in CKD patients under maximal tolerated inhibition of renin-angiotensin system: a prospective observational study

**DOI:** 10.1186/1471-2369-13-150

**Published:** 2012-11-20

**Authors:** Luca De Nicola, Giuseppe Conte, Domenico Russo, Antonio Gorini, Roberto Minutolo

**Affiliations:** 1 Nephrology Departments at Second University, Napoli, Italia; 2 University Federico II, Napoli, Italia; 3 S. Giovanni Evangelista Hospital, Tivoli, Italy; 4 Cattedra di Nefrologia - Dip. Gerontologia, Geriatria, Mal. Metabolismo, Seconda Università di Napoli, Facoltà di Medicina, Piazza Miraglia, 80131, Napoli, Italia

**Keywords:** Angiotensin converting enzyme inhibitor, Angiotensin II receptor blocker, Renin inhibitor, Paricalcitol, Chronic kidney disease, Proteinuria

## Abstract

**Background:**

Whether paricalcitol (PCT) reduces proteinuria in the presence of intensified inhibition of Renin-Angiotensin-System (RAS) is poorly studied. We evaluated the antiproteinuric effect of PCT in non-dialysis chronic kidney disease (CKD) patients with proteinuria greater than 0.5 g/24 h persisting despite anti-RAS therapy titrated to minimize proteinuria in the absence of adverse effects.

**Methods:**

Forty-eight CKD patients were studied in the first six months of add-on oral PCT (1 mcg/day) and three months after drug withdrawal.

**Results:**

Males were 87.5%, age 63 ± 14 yrs, systolic/diastolic blood pressure (BP) 143 ± 22/78 ± 11 mmHg, eGFR 29.7 ± 14.5 mL/min/1.73 m^2^, diabetes 40%, and cardiovascular disease 38%. At referral in the center (28 months prior to study baseline), proteinuria was 2.44 (95% CI 1.80-3.04) g/24 h with 6 patients not receiving any anti-RAS and 42 treated with a single agent, at low dosage in most cases. At study baseline, twenty patients were under 2–3 anti-RAS drugs while twenty-eight received 1 agent at full dose and proteinuria resulted to be reduced versus referral to 1.23 g/24 h (95%CI 1.00-1.51). Six months of add-on PCT significantly decreased proteinuria to 0.61 g/24 h (95%CI 0.40-0.93), with levels less than 0.5 g/24 h achieved in 37.5% patients, in the absence of changes of BP and GFR. Proteinuria recovered to basal value after drug withdrawal. The extent of antiproteinuric response to PCT was positively associated with diabetes, eGFR and daily Na excretion (R^2^ = 0.459, P < 0.0001). PTH decreased from 201 (IQR 92–273) to 83 (IQR 50–189) pg/mL.

**Conclusions:**

In CKD patients, add-on PCT induces a significant reduction of proteinuria that is evident despite intensified anti-RAS therapy and larger in the presence of diabetes, higher GFR and unrestricted salt intake.

## Background

Proteinuria is a well recognized surrogate outcome for long-term prognosis in patients with chronic kidney disease (CKD) [1]. The magnitude of proteinuria predicts in fact renal and cardiovascular (CV) events [1-3], and, more important, reduction is associated with slowing of CKD progression and CV protection [4,5].

Inhibition of the renin-angiotensin system (RAS) is the cornerstone of treatment in proteinuric patients with the effect being largely independent of blood pressure (BP) control [1,4-6]. However, the high complexity of the system with multiple-level escape mechanisms prevents adequate suppression [7,8]. Indeed, monotherapy with either angiotensin converting enzyme inhibitor (ACEi) or angiotensin receptor blocker (ARB) decreases proteinuria by about 30% [9]. Multiple blockade of RAS with either two or three drugs allows an additional decrement of proteinuria which is however still limited and heterogeneous, with many patients being left with significant proteinuria [10,11]. Therefore, novel antiproteinuric strategies aimed at attaining remission of proteinuria (<0.5 g/24 h) are sought.

In the last decade, experimental evidence has been collected on an important renoprotective role of vitamin D hormone and its analogs [12,13]. Furthermore, randomized clinical trials in non-dialysis CKD have evidenced that paricalcitol (PCT), the low-calcemic vitamin D analog currently used in Europe to prevent and treat secondary hyperparathyroidism, also acts as antiproteinuric agent when added to standard anti-RAS therapy with a single agent [14-18]. However, evidence on the antiproteinuric effect of PCT in the presence of intensified anti-RAS therapy is limited [17]. This question is critical for daily nephrology practice because intensified anti-RAS therapy, while being possibly contraindicated in patients with absent or low-degree proteinuria, is warranted in patients with proteinuric CKD treated in renal clinics [11,19,20].

Aim of this study was to verify the antiproteinuric response to PCT in adult CKD patients with residual proteinuria persisting after maximal tolerated anti-RAS therapy.

## Methods

This is a prospective observational study conducted in CKD patients regularly attending three nephrology outpatient clinics. The study was approved by the Institutional Review Board of the participating centers (L.D.N., G.C. and R.M.: AOU-Second University of Naples; A.G.: ASL ROMA G; D.R.: AOU-University Federico II of Naples) and all patients gave informed consent.

### Characteristics of centers

The three participating centers share a protocol for management of CKD patients. Patients are always seen by the same nephrologist since the first visit in the center (referral visit). Office systolic and diastolic BP target is <130 and <80 mmHg, respectively. If at referral the patient is identified as having white coat hypertension (WCH), that is, BP ≥130/80 in office and normal (<125/75 mmHg) 24 h ambulatory BP (ABP) [21,22], office BP target is less restrictive being equal to 130/80 mmHg. In the three centers, ABP is performed by using Spacelabs 90207 monitors.

All patients receive personalized dietary regimens to limit dietary salt (<6 g NaCl/day) and protein (≤0.8 g/kg body wt/day), and to avoid food and beverages containing high amounts of potassium. They also periodically undergo to serum bicarbonate testing to diagnose and treat metabolic acidosis. Compliance to drugs is evaluated at each visit in the clinic by means of specific questions on assumption of pharmacological therapy directed to the patient and family members. Specifically, physicians ask the number of times the patient had not taken the prescribed medications in the last two weeks; the patient is identified as poorly compliant to therapy, and therefore excluded from any study, if the missing rate is ≥20%.

### Intensification of anti-RAS therapy

In the three centers, anti-RAS therapy is titrated to achieve remission of proteinuria (<0.5 g/24 h) in the absence of adverse effects (symptomatic hypotension or systolic blood pressure <100 mmHg, acute eGFR decrease >30% or serum potassium >5.5 mmol/L).

Participating nephrologists first administer a single anti-RAS agent (either ACEi or ARB), titrated to the full dose defined according to the manufacturer’s recommendations in Italy; if proteinuria still persists above the target we combine ACEi with ARB and/or add Aliskiren (this drug is not used anymore in combination with other anti-RAS drugs according to the recommendations of the European Medicines Agency and FDA released on February and April 2012, respectively). Anti-aldosterone drugs are used as additional antiproteinuric agents only when GFR is ≥ 60 mL/min/1.73 m^2^. In the case of low BP levels, antihypertensive drugs other than anti-RAS are downtitrated.

### Selection criteria

From September 2010 to January 2011, we selected consecutive adult CKD patients under nephrology care from at least six months in the three participating centers with eGFR <60 mL/min/1.73 m^2^ (no dialysis-no transplant), proteinuria >0.5 g/24 h on two consecutive visits (30 days apart) and under intensified anti-RAS therapy unchanged from ≥3 months. Exclusion criteria were steroid/ immunosuppressive treatment or eGFR change >30% in the past 3 months, PTH levels <20 pg/mL, serum phosphorus >5.0 mg/dL, serum calcium (adjusted for albumin) >10.0 mg/dL, active malignancy.

### Add-on paricalcitol

Data were collected at baseline (prior to first administration of PCT), during administration of oral PCT (after 3 and 6 months), and three months after PCT withdrawal. PCT was administered at starting dosage of 1 mcg/day (8:00–9:00 a.m.); this dosage was chosen as it is not associated with excessive decline of parathyroid hormone (PTH) levels in most patients [16,17]. During the study, investigators were allowed to modify the dosage of PCT on the basis of PTH and proteinuria levels.

At each visit of the study, nephrologist performed physical examination and BP measurement. Two consecutive 24 h-urine collection were required and results averaged. Urine collections were considered inaccurate, and repeated, if the value of measured creatinine excretion rate fell outside the normal range. Blood and urinary samples were analyzed by the in-hospital laboratory. GFR was estimated by the four-variable MDRD equation.

### Statistics

Variables are reported as mean and standard deviation (SD) or as median and interquartile range (IQR). Proteinuria values are expressed as geometric mean and 95% confidence interval (CI) because of their positively skewed distribution. Means are compared by paired or unpaired Student t test and McNemar or chi-square test for categorical variables. ANOVA for repeated measures with Bonferroni as post-hoc test are also used where appropriate. Multivariate linear regression analysis was also performed to identify the predictors of antiproteinuric response to PCT (percent change at month 6 of 24-h proteinuria from baseline). A two-tailed P-value <0.05 is considered significant. Data are analyzed using SPSS 12.0 (SPSS Inc, Chicago, IL, USA).

## Results and discussion

### Results

We studied 48 Caucasian patients out of the 76 identified by inclusion criteria; twenty-eight patients were in fact excluded because of recent immunosuppressive treatment (n = 8), acute GFR change (n = 8), poor compliance to therapy (n = 7), low PTH (n = 5).

At first visit in the center (referral), performed 28 months on median (IQR 16–40) prior to add-on PCT (study baseline), patients had eGFR 38 ± 18 mL/min/1.73 m^2^ and proteinuria 2.44 (95% CI 1.80-3.04) g/24 h. Office BP was 148 ± 18/83 ± 13 mmHg while 24 h ABP was 133 ± 17/76 ± 11 mmHg, with 43% patients being identified as having WCH. At referral, the mean number of antihypertensive drugs was 2.8 ± 1.0; forty-two patients were treated with a single anti-RAS drug, at low dosage in 90% cases, while the remaining six patients were anti-RAS naïve. In the first months after referral visit, four patients had been treated with immunosuppressive agents.

Table 1 shows the main basal characteristics of patients. Patients showed a high-risk profile as evidenced by the advanced age, high BMI and the large prevalence of diabetes and CV disease. Adherence to prescribed low salt diet (Na excretion ≤100 mmol/24 h) was low (19%). The increase in the number of BP lowering drugs, observed at baseline versus referral visit, was mainly due to the larger use of anti-RAS agents. At baseline, in fact, twenty patients were under multiple blockade of RAS; they received either ACEi + ARB (n = 11) or ARB + Aliskiren (n = 1) or ACEi + Aliskiren (n = 1) or ACEi + ARB + Aliskiren (n = 7). Intensification of anti-RAS therapy was also obtained in the twenty-eight patients administered a single anti-RAS agent; at referral, in fact, these drugs were not used in four or used at low dosage in the remaining twenty-four patients while at baseline all patients were treated with anti-RAS at full dose. In these 28 patients, combined therapy was considered unfeasible because, during the previous follow up in the center, adding the second anti-RAS agent had been associated with documented acute GFR decline (n = 8), hypotension (n = 7), hyperkalemia (n = 13). As compared with patients under combined anti-RAS therapy, those receiving anti-RAS monotherapy were older (69 ± 11 vs 55 ± 14 years) and had lower levels of eGFR (26 ± 12 vs 35 ± 17 mL/min/1.73) (P < 0.05 for both).

**Table 1:** Basal characteristics of patients, overall and by extent of proteinuria reduction after six months of paricalcitol

	**Overall**	**Good Responders(ΔUprot≥30%)**	**Poor Responders(ΔUprot<30%)**	**P**
N	48	24	24	
Age (years)	63 ± 14	64.1 ± 11.5	62.7 ± 16.9	0.735
Gender (M:F)	42:6	22:2	20:4	0.666
Body Mass Index (Kg/m^2^)	27.6 ± 4.4	27.9 ± 3.8	27.2 ± 5.1	0.593
Diabetes (% pts)	19 (39.6)	14 (58.3)	5 (20.8)	0.017
Previous CV events (% pts)	18 (37.5)	11 (45.8)	7 (29.2)	0.371
Renal Disease (pts)	DN (12), GN (10) HN (13), APKD (4) Pyelonephritis (2) Nephrectomy (1) Unknown (6)	DN (9), GN (6), HN (4), APKD (2) Pyelonephritis (1) Nephrectomy (0) Unknown (2)	DN (3), GN (4), HN (9), APKD (2) Pyelonephritis (1) Nephrectomy (1) Unknown (4)	0.322
25-OH vitamin D (ng/mL)	18.8 ± 10.0	19.9 ± 8.7	17.6 ± 11.2	0.433
eGFR (mL/min/1.73 m^2^)	29.7 ± 14.5	35.5 ± 16.0	23.9 ± 10.1	0.004
Serum albumin (g/dL)	4.1 ± 0.4	4.0 ± 0.5	4.2 ± 0.4	0.241
Proteinuria (g/day)	1.23 (1.00-1.51)	1.14 (0.81-1.61)	1.32 (1.02-1.71)	0.501
UNaV (mEq/day)	161 ± 63	183 ± 60	141 ± 52	0.012
Systolic BP (mmHg)	143 ± 22	146 ± 19	141 ± 24	0.453
Diastolic BP (mmHg)	78 ± 11	78 ± 11	78 ± 12	0.980
Antihypertensive Drugs (n)	3.6 ± 1.4	3.3 ± 1.4	3.8 ± 1.4	0.224
Anti-RAS per patient (n)	1.6 ± 0.7	1.5 ± 0.7	1.6 ± 0.8	0.848
Furosemide (%)	24 (50.0)	14 (58.3)	10 (41.7)	0.387
Furosemide dose (mg/d)	63 ± 30	60 ± 32	64 ± 31	0.742
CCB (%)	25 (52.1)	13 (54.2)	12 (50.0)	1.00
Beta Blocker (%)	27 (56.3)	17 (70.8)	10 (41.7)	0.080

Data are mean ± SD or percentage or geometric mean and (95% confidence interval). CV, cardiovascular; DN, diabetic nephropathy; GN, glomerulonephritis; HN, hypertensive nephropathy; APKD, autosomal polycystic kidney disease; eGFR, 4-variable MDRD estimated GFR; BP, blood pressure; RAS, renin angiotensin system; CCB Calcium Channel Blocker.

As reported in Table 2, add-on PCT was associated with a progressive decline in PTH levels that, however, did not decrease below the lower limit of normal range (20 pg/mL) in any patient. No significant change of alkaline phosphatase (ALP) levels was observed, with only two patients at month 3 and one patient at month 6 showing ALP levels below the lower limit of normal range (40 IU/L). Serum calcium and phosphate remained within normal range in all patients but one that had a single episode of hyperphosphatemia (6.4 mg/dL) at month 3 due to excessive phosphorus intake. In this patient, PCT was temporarily withdrawn and re-started at 1 mcg/day within one month after successfully reinforcing dietary advices and administering a phosphate binder. Binders were constantly administered throughout follow up in seven patients.

**Table 2:** Changes of main parameters during Paricalcitol (PCT) in the whole cohort (n = 48) and three months after withdrawal (n = 42)

	**Basal**	**Month 3**	**Month 6**	**PCT withdrawal**
SBP/DBP (mmHg)	143 ± 22/78 ± 11	137 ± 15/78 ± 10	138 ± 17/79 ± 10	134 ± 16/77 ± 9
eGFR (mL/min/1.73 m^2^)	29.7 ± 14.5	27.3 ± 15.5	27.5 ± 16.2	26.9 ± 15.3
Serum potassium (mmol/L)	4.6 ± 0.7	4.8 ± 0.7	4.8 ± 0.6	4.7 ± 0.6
PTH (pg/mL)	201 (92–273)	140 (64–226)*	83 (50–189)*	111 (74–184)*
ALP (IU/L)	155 ± 96	148 ± 86	143 ± 93	135 ± 95
Serum Calcium (mg/dL)	9.3 ± 0.6	9.3 ± 0.5	9.4 ± 0.4	9.3 ± 0.6
Serum Phosphate (mg/dL)	3.9 ± 0.7	3.9 ± 0.8	3.9 ± 0.7	3.9 ± 0.7
Proteinuria (g/24 h)	1.23 (1.00-1.51)	0.85 (0.59-1.21)*	0.61 (0.40-0.93)*	1.12 (0.86-1.44)
UNaV (mmol/24 h)	161 ± 63	144 ± 55	149 ± 65	157 ± 66

Data are mean ± SD or median and interquartile range (PTH) or geometric mean and 95% confidence interval (proteinuria). SBP/DBP, systolic/diastolic blood pressure; eGFR, 4-variable MDRD estimated GFR; ALP, Alkaline Phosphatase; UNaV, 24 h urinary Na excretion. *P < 0.05 vs basal.

Add-on PCT induced a progressive decrease of proteinuria (Table 2). Remission of proteinuria to values <0.5 g/24 h was achieved in 14.6% at month 3, and in 37.5% by month 6 (P = 0.007 vs month 3). These results were obtained in the presence of an unchanged antihypertensive therapy (mean number from baseline to month 6 was 3.6 ± 1.4, 3.6 ± 1.4 and 3.4 ± 1.4, respectively) and a small reduction in the number of anti-RAS (1.6 ± 0.7, 1.4 ± 0.8 and 1.4 ± 0.8, respectively). The median reduction of proteinuria after six months of PCT was 32% (IQR 11–52). No correlation was found between percentual change in proteinuria and systolic BP (r = 0.146, P = 0.324).

Table 1 reports the basal characteristics of good and poor responders to PCT, as defined according to the extent of proteinuria reduction around the median value (≥30% and <30%, respectively). Median reduction of proteinuria was 57% (IQR: 46–72) in good responders and 11% (from −8 to 22) in poor responders. Good responders showed greater prevalence of diabetes, and higher eGFR and 24 h urinary sodium excretion. Multiple linear regression analysis of antiproteinuric response as continuous variable (model summary: R^2^ = 0.459, P < 0.0001) confirmed the greater antiproteinuric effect in the presence of diabetes (P = 0.022), higher eGFR (P = 0.004) and higher daily Na excretion (P = 0.005) while association with age and gender was not significant.

The first dose of PCT (1 mcg/day) did not change throughout the six months of follow up in 40/48 patients, halved in three patients (low PTH or gastrointestinal intolerance), and doubled in five (increasing PTH and/or proteinuria).

**Figure 1:**
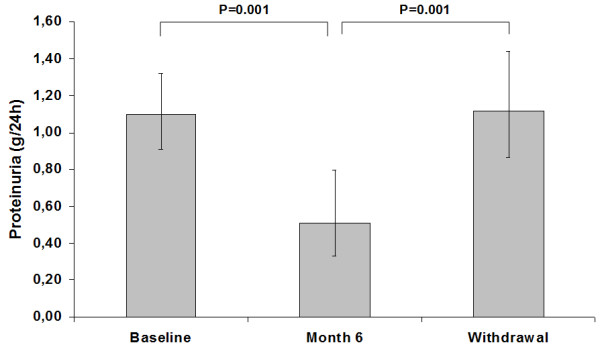
**Proteinuria levels measured in 42 patients before (baseline), after add-on Paracalcitol (Month 6), and after three-month drug withdrawal.** Data are geometric mean and 95% confidence interval. See text (Results) for missing patients.

To verify the dependence of proteinuria reduction on PCT, we evaluated the change of proteinuria three months after drug withdrawal. This analysis was not possible in six patients because, after month 6, three of them started hemodialysis, two had a flare of underlying glomerulonephritis that required immunosuppressive therapy, and one moved to other town. In the 42 patients undergoing PCT withdrawal, proteinuria recovered to baseline (Figure 1). In these patients, median PTH was 206 (102–270) pg/mL at baseline, 90 (51–188) pg/mL at month 6 and 111 (74–184) pg/mL after withdrawal (P < 0.05 for month 6 and withdrawal versus baseline).

### Discussion

The co-existence of proteinuria >0.5 g/24 h and GFR <60 mL/min/1.73 m^2^ identifies a subgroup of CKD patients which carries the highest cardio-renal risk [1-3]. In these patients, minimizing RAS activity is a main target of treatment [1,3,6]. Such a subgroup of CKD patients was the object of the current study. Enrolled subjects were in fact encumbered by multiple risk factors and significant proteinuria despite intensified anti-RAS treatment. This therapeutic feature represents the main difference with the previous studies on antiproteinuric effect of PCT where patients did not consistently undergo intensification of anti-RAS therapy prior to add-on PCT [14-17]. In our cohort, the preliminary intensification of anti-RAS therapy during the interval between referral and study baseline (two years on median) is testified by the marked decrease of proteinuria in the absence of major changes of antihypertensive therapy other than anti-RAS. As a consequence of the predominant use of anti-RAS versus other drugs, only a mild decline of BP values from the referral visit was observed. The large prevalence of WCH (43%), that is expected in tertiary nephrology care [23], further restrained participating nephrologists from intensifying antihypertensive agents other than anti-RAS. Of note, in this observational study in outpatient clinics, we enrolled patients under maximal tolerated anti-RAS therapy. In this regard, it is noteworthy that patients treated with a single anti-RAS, that were older and with more advanced CKD, had documented side effects when implementation of combined treatment was attempted. Under the peculiar condition of intensified anti-RAS therapy, add-on PCT induced a 32% reduction of proteinuria, with a substantial portion of patients (38%) showing remission of proteinuria (<0.5 g/24 h).

Assessment of underlying pathophysiological mechanisms goes beyond the original purpose of the study. We hypothesize that the additive antiproteinuric effect of PCT may be at least in part related to more effective RAS inhibition. Indeed, experimental data converge to indicate that PCT blunts the compensatory increase of renin synthesis secondary to the disruption of the feedback inhibition loop occurring during chronic administration of anti-RAS agents [12,13,24]. Therefore, the detrimental effects on the kidney of renin, that can occur also independently from AII [7], may be limited in the course of add-on PCT therapy. Nevertheless, anti-inflammatory effects, direct and indirect (mediated by suppression of intrarenal RAS activity), as well as lessening of podocyte injury, cannot be excluded [12,13,25,26].

The extent of antiproteinuric effect of PCT was greater in the presence of higher GFR, diabetes and higher salt intake (Table 1). These associations persisted when considering the proteinuria change as continuous variable in the multiple regression analysis adjusted for age and gender. The influence of GFR is expected, being conceivably related to the amount of irreversible proteinuria that increases in parallel with advancing nephrosclerosis [2]. Therefore, in the specific context of this study including patients with GFR <60, the more is preserved renal function the greater is the reduction of proteinuria. In this regard, recent studies have disclosed that also less expensive interventions such as nutritional vitamin D repletion or administration of calcitriol, that is a less potent and more calcemic vitamin D analog [27], reduce proteinuria in patients with milder degrees of renal disease, such as microalbuminuric diabetic nephropathy with moderate GFR impairment and IgA nephropathy with close-to-normal GFR [28,29].

Of greater interest is the larger antiproteinuric effect of PCT evidenced in diabetics; the higher response may be related to the greater activity of intrarenal RAS [30,31], and/or to the specific positive effects of PCT on glomerular barrier [26]. Interestingly, recent studies have also evidenced an association between decrements of albuminuria and urinary TGF-β in type 2 diabetics treated with cholecalciferol [28].

On the other hand, more puzzling is the positive relationship between antiproteinuric response and salt intake. Indeed, it is well known that high salt intake blunts the beneficial effects of anti-RAS therapy [32,33]. As in the current study, also the VITAL trial showed enhanced antiproteinuric effect of add-on PCT in diabetic CKD patients under standard anti-RAS therapy when salt intake was high [17]. We obtained similar data when we evaluated the antiproteinuric effect of aliskiren given on the top of dual RAS blockade [11]. On this basis, we can therefore hypothesize that the greater antiproteinuric effect of PCT in patients at higher sodium intake could be explained by the suboptimal antiproteinuric efficacy of anti-RAS under these conditions. Conversely, when patients adhere to sodium restriction, the antiproteinuric response to anti-RAS is already elevated and the antiproteinuric effect of add-on PCT is therefore limited. Independent of underlying mechanism, the finding is of great clinical interest as the majority of patients are not compliant to salt restriction even if followed in renal clinics [2].

The significant antiproteinuric effect of PCT was not impaired by major adverse effects, thus extending to this subgroup of high-risk and poly-treated patients the favorable safety profile of PCT previously reported [13-18]. PTH and ALP remained in the normal range in most patients. This finding supports the experimental evidence that low-dose PCT, given in the absence of exogenous calcium loading, is not associated to excessive suppression of bone turnover [34].

Interpretation of results is limited by the small sample size and the absence of control group; nevertheless, the remarkable antiproteinuric response and the recovery of proteinuria to baseline after three months of drug withdrawal support the validity of our observations. As regard the recovery of proteinuria after drug withdrawal, however, we cannot exclude some degree of escape of proteinuria which is a not a uncommon finding when inhibition of RAS is intensified (7,8,11). Furthermore, more prolonged follow up is required to verify efficacy and safety of this approach over the long-term. Finally, we did not assess systemic RAS activity. While it precludes from gaining insights into the mechanism(s) of the antiproteinuric effects of add-on PCT, the antihypertensive poly-therapy and the outpatient setting of the study, with the consequent uncontrolled external balance of sodium, prevent any meaningful evaluation of this system in our patients. However, it is well known that intrarenal rather than systemic RAS acts as the major control mechanism [7,8].

## Conclusions

This study suggests that in patients with low GFR and persisting proteinuria despite maximal tolerated anti-RAS therapy: (1) add-on PCT safely allows a reduction of proteinuria with a remarkable achievement of levels <0.5 g/24 h in more than one-third of cases, (2) antiproteinuric response to PCT is greater in diabetics, patients with higher GFR and in the presence of unrestricted salt intake.

PCT therefore represents a potential additional therapeutic option to be considered when intensified anti-RAS treatment is not completely efficacious or unfeasible because of adverse effects. Whether the antiproteinuric effect of PCT translates into improvement of prognosis of these high-risk patients is worth investigating.

## Abbreviations

ABP: 24 h Ambulatory blood pressure; ACEi: Angiotensin converting enzyme inhibitor; ALP: Alkaline phosphatase; ARB: Angiotensin receptor blocker; BMI: Body mass index; BP: Blood pressure; CKD: Chronic kidney disease; CV: Cardiovascular; eGFR: Estimated glomerular filtration rate; IQR: Interquartile range; MDRD: Modification of diet in renal disease; PCT: Paricalcitol; PTH: Parathyroid hormone; RAS: Renin-Angiotensin-System; SD: Standard deviation; VITAL: Selective vitamin D receptor activator for Albuminuria lowering; WCH: White coat hypertension.

## Competing interests

There is no conflict of interest on this work.

## Authors’ contributions

LDN and RM had full access to all the data in the study and takes responsibility for the integrity of the data and the accuracy of the data analysis. All Authors gave the final approval of the submitted version of the paper. Study concept and design: LDN, RM. Acquisition of data: LDN, GC, AG, RM, DR. Analysis and interpretation of data: LDN, RM. Drafting of the manuscript: LDN. Critical revision of the manuscript: GC, AG, RM, DR. Study supervision: LDN, GC, AG, RM, DR. All authors read and approved the final manuscript.

## Authors’ information

LDN: Associate Professor of Nephrology, Second University of Naples, Italy.

GC: Full Professor of Nephrology, Second University of Naples, Italy.

DR: Associate Professor of Nephrology, University Federico II of Naples, Italy.

AG: Consultant Nephrologist, Nephrology and Dialysis Unit, S. Giovanni Evangelista Hospital, Tivoli, Italy.

RM: Aggregate Professor of Nephrology, Second University of Naples, Italy.

All Authors are members of the Italian Society of Nephrology.

## Transparency declarations

L.D.N. has received consulting fee from Amgen and Roche and honoraria for lectures for Abbott, Amgen, Roche.

G. C. has received honoraria for lectures for Amgen, Roche.

D.R. received honoraria for lectures for Roche, Abbott, Genzyme, Amgen.

A.G. has received consulting fee from Abbott.

R.M. has received consulting fee from Roche and honoraria for lectures for Abbott, Amgen, Roche.

## Pre-publication history

The pre-publication history for this paper can be accessed here:

http://www.biomedcentral.com/1471-2369/13/150/prepub
